# Cronkhite-Canada Syndrome: A Rare Case of Chronic Diarrhea With Ectodermal Changes

**DOI:** 10.7759/cureus.29298

**Published:** 2022-09-18

**Authors:** Natapat Chaisidhivej, Monthira Maneerattanaporn, Ananya Pongpaibul, Angkawipa Trongtorsak, Jami Kinnucan

**Affiliations:** 1 Department of Medicine, Einstein Medical Center Philadelphia, Jefferson Health, Philadelphia, USA; 2 Division of Gastroenterology, Department of Medicine, Faculty of Medicine Siriraj Hospital, Mahidol University, Bangkok, THA; 3 Department of Pathology, Faculty of Medicine Siriraj Hospital, Mahidol University, Bangkok, THA; 4 Internal Medicine, AMITA Health Saint Francis Hospital, Evanston, USA; 5 Division of Gastroenterology and Hepatology, Department of Internal Medicine, Mayo Clinic, Jacksonville, USA

**Keywords:** alopecia, onychodystrophy, cutaneous hyperpigmentation, gastrointestinal polyposis, cronkhite-canada syndrome (ccs)

## Abstract

Cronkhite-Canada syndrome (CCS) is a rare cause of chronic diarrhea and malabsorption where patients develop multiple polyps throughout the gastrointestinal (GI) tract, accompanied by ectodermal changes. Due to its rarity, early detection and diagnosis are challenging for physicians, inevitably leading to high mortality. CCS patients have a higher prevalence of GI cancer compared to the general population. Therefore, a follow-up endoscopy is necessary. We report a new case of CCS in an 85-year-old male who presented with chronic watery diarrhea, weight loss, and skin changes including alopecia, nail dystrophy, and hyperpigmentation. Laboratory results showed anemia and hypoalbuminemia. He underwent an endoscopy that found diffuse edematous polyposis in the stomach, duodenum, terminal ileum, and large intestine. The biopsy result confirmed the diagnosis of CCS. The patient received supportive treatment with total parenteral nutrition with improvement in his symptoms. He was placed on corticosteroid taper and azathioprine upon discharge. At the one-year follow-up, he was found in endoscopic remission.

## Introduction

Cronkhite-Canada syndrome (CCS) is a very rare non-familial gastrointestinal (GI) disease manifested by diffuse GI hamartomatous polyposis together with ectodermal abnormalities, including alopecia, onychodystrophy, and hyperpigmentation [1]. Although its exact etiology is unknown, CCS is believed to be related to the autoimmune process [2]. Most case reports have been reported from Asian countries [3]. It has been suggested that manifestations are a result of a malabsorptive process due to protein-losing enteropathy leading to significant weight loss and malnutrition [2,4]. The mortality rate is high because of the difficulty in diagnosis and delay in treatment [2-4]. Therefore, early detection is crucial. CCS may also be associated with an increased risk of intestinal malignancy, for which surveillance follow-up is needed [2].

## Case presentation

**Figure 1:**
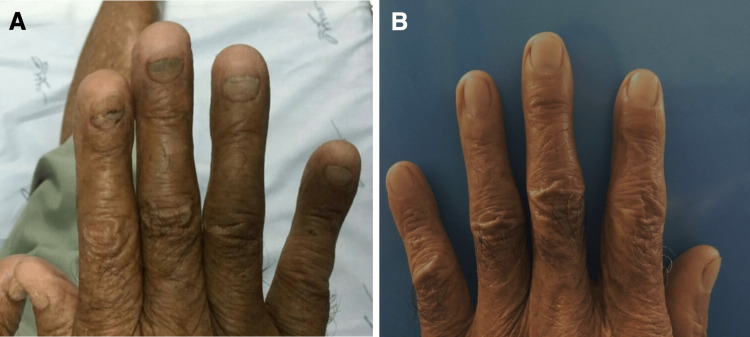
Onychodystrophy. (A) Before remission. (B) After remission.

**Figure 2:**
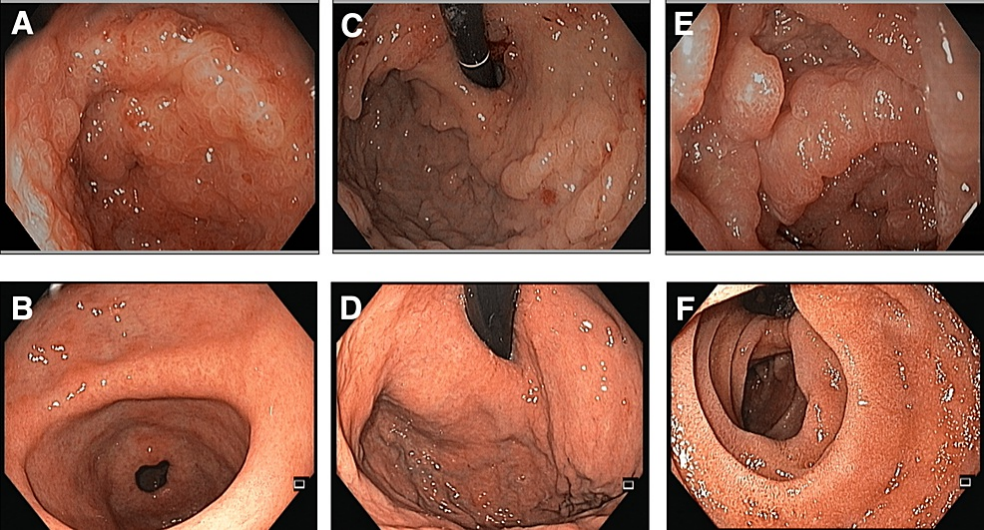
Endoscopy comparing between before and after remission. (A, B) Antrum. (C, D) Fundus. (E, F) Duodenum.

**Figure 3:**
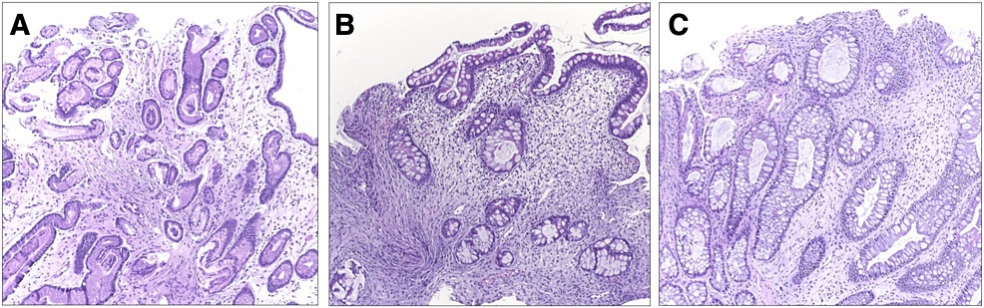
Histological findings showing edematous changes of the mucosa and mixed inflammatory infiltration in the lamina propria, together with cystically dilated intestinal crypts along the digestive tract. (A) Stomach. (B) Duodenum. (C) Colon.

An 85-year-old man with no medical history presented with watery diarrhea and an unexplained 30-pound weight loss over three months accompanied by loss of appetite, anorexia, and loss of taste sensation. Just before the presentation, he was hospitalized for severe hypovolemia requiring intravenous (IV) fluid hydration. On physical examination, he had alopecia, nail dystrophy (Figure 1), and hyperpigmentation of the skin on his face and bilateral upper extremities. He was noted to have pale conjunctiva and generalized pitting edema; however, his abdominal examination was normal. The laboratory results showed hemoglobin 6.9 g/dL, leukocyte count 9.4 × 10^9^/L, platelet count 135 × 10^9^/L, and serum albumin 2.1 g/dL. His liver function tests and other blood chemistry panels were normal (Table 1). Stool examination was positive for *Blastocystis hominis* and *Clostridium difficile*. Endoscopy found diffuse edematous polyposis at the stomach, duodenum, terminal ileum, and colon (Figure 2). Histology revealed focally eroded edematous mucosa with cystically dilated foveolar hyperplasia. The lamina propria contained mixed inflammatory cells (Figure 3). Based on the constellation of findings, the patient was given a diagnosis of CCS.

**Table 1:** Laboratory results on presentation and one-year follow-up.

	First presentation	One-year follow-up
Hemoglobin (g/dL)	6.9	13.4
Hematocrit (%)	21.5	39.6
White blood cell count (×10^9^/L)	9.4	7.4
Platelet count (×10^9^/L)	135	270
Creatinine (mg/dL)	0.7	0.8
Total protein (g/dL)	4.6	7.0
Albumin (g/dL)	2.1	4.0
Aspartate transaminase (U/L)	21	14
Alanine transaminase (U/L)	8	11

The patient received metronidazole (for bacterial infections) and supportive treatment along with total parenteral nutrition supplementation for a total course of 10 days. The patient was discharged home on a prednisone taper and maintenance therapy with azathioprine. The patient responded well to the treatment, and his symptoms were improving after discharge. A one-year follow-up endoscopy after the presentation was endoscopically normal with no polyps, and histology revealed evidence of chronic inactive gastritis with intestinal metaplasia. The laboratory results showed improvement in anemia and nutritional status.

## Discussion

Leonard Cronkhite and Wilma Canada first reported two interesting cases of uncommon diarrhea in 1955, which later bear their name, CCS [1]. Due to its rarity, most CCS studies are documented as case reports, with the majority reported from Japan [3]. The mean age of onset is 63.5 years with a male-to-female ratio of 1.84:1 [2]. The pathogenesis of the disease is not clear but believed from an inflammatory process in response to anti-inflammatory and immunosuppressive medications [2]. Many studies have shown that histopathologic findings of CCS patients are associated with immunoglobulin G4 (IgG4) expression; meanwhile, several studies have shown an association between CCS and IgG4-related disorders [4,5]. In addition, there are several reports showing that CCS is associated with autoimmune diseases such as membranous nephropathy, and some CCS patients have a positive antinuclear antibody test [4,6].

Affected patients usually develop chronic diarrhea with significant weight loss, abdominal pain, and dysgeusia. The associated triad of ectodermal changes is simultaneously found, including alopecia, hyperpigmentation, and onychodystrophy [1,7]. Prolonged diarrhea and protein-losing enteropathy inevitably lead to severe protein-calorie malnutrition. The majority have anemia and zinc deficiency [2]. The typical endoscopic finding is diffuse sessile polyposis throughout the GI tract with esophageal sparing. Histologically, the polyps are characterized as non-neoplastic, inflammatory, and hamartomatous type, with cystic dilation of the gland without atypia [8]. However, it is sometimes difficult to distinguish CCS from other polyposis diseases such as juvenile polyposis syndrome in that they have some similarities in both endoscopic and histopathologic findings.

Despite the high morbidity, proper medical treatment contributes to improvement in the course of the disease and outcomes [2]. The initial step in management is supportive management and parenteral nutritional support. Like in our case, with early detection, the patient received supportive treatment promptly leading to rapid recovery and a short hospitalization period in comparison with other case reports [9]. Most patients are steroid responsive, and corticosteroids are important for induction of remission with taper [2,4,9]. Patient relapse usually occurs in a tapering period of corticosteroids, hence, immunomodulators, especially thiopurines, have been used in the maintenance of remission, as in our case [4]. The goal of treatment is both clinical and endoscopic remission which reflects long-term response [2-4]. Cyclosporine is used in many cases of steroid-refractory CCS which can achieve remission; however, there is no systematic report showing the long-term efficacy of cyclosporine [10,11]. In addition, an anti-tumor necrosis factor, infliximab, is used as initial treatment which shows some promising outcomes [12,13]. Patients who fail to improve with pharmacological treatment may be considered for surgical management [2-4,7].

There have been several reports of patients with CCS developing neoplastic transportation of polyps. The prevalence of gastric and colon cancer in CCS patients is about 10-25% [2]. The most common sites are the sigmoid colon and rectum [14]. Annual endoscopic surveillance is recommended to confirm ongoing remission, as endoscopic remission is associated with less death from disease complications and decreased risk of developing GI cancer [2].

## Conclusions

Although the rarity of CCS results in difficulty in diagnosis, early detection is significant as this can prevent complications and decrease the mortality rate. It is necessary to find effective methods to help physicians differentiate CCS from other conditions to avoid delay in diagnosis which may worsen the treatment outcomes. The follow-up endoscopy is significant as endoscopic remission is associated with decreased cancer risk.
